# Supplementary material for: Seamless Tube-Type Heater with Uniform Thickness and Temperature Distribution Based on Carbon Nanotubes Aligned by Circumferential Shearing
Source: Materials (Basel). 2019 Oct 9;12(20):3283. doi: 10.3390/ma12203283 (PMC6829887; doi:10.3390/ma12203283)
Supplement: Supplementary file 1 [file materials-12-03283-s001.pdf]

materials

Supplementary Materials

Article

Seamless Tube-Type Heater with Uniform Thickness and Temperature Distribution Based on Carbon Nanotubes Aligned by Circumferential Shearing

Yoonchul Sohn [1], Dongearn Kim [2,†], Sung-Hoon Park [3,*] and Sang-Eui Lee [4,*,†]

[1] Department of Welding & Joining Science Engineering, Chosun University, 309 Pilmun-daero, Dong-gu, Gwangju 61452, Korea; yoonchul.son@chosun.ac.kr
[2] Molds & Dies Technology Group, Korea Institute of Industrial Technology, 7-47 Songdo-dong, Yeonsoo-gu, Incheon 21999, Korea; kdu0517@kitech.re.kr
[3] Department of Mechanical Engineering, Soongsil University, 369 Sangdo-ro, Donjak-gu, Seoul 06978, Korea
[4] Department of Mechanical Engineering, Inha University, Inha-ro 100, Michuhol-gu, Incheon 22212, Korea
[†] Materials Research Center, Samsung Advanced Institute of Technology, Samsung-ro 130, Suwon 16678, Korea (previous workplace)
[*] Correspondence: selee@inha.ac.kr (S-.E.L.); leopark@ssu.ac.kr (S-.H. P.); Tel.: +82-32-860-7377 (S-.E.L.); +82-2-828-7021 (S-.H. P.)

materials MDPI

Surface morphology	Roller speed (v_1-v_2)
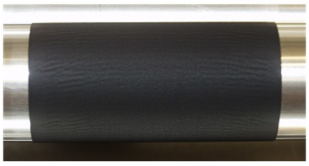	50-45
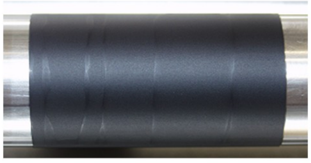	100-45
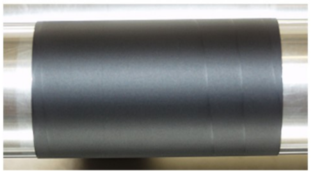	200-60
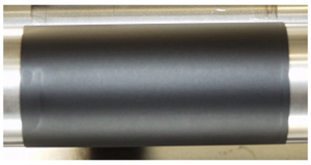	300-60

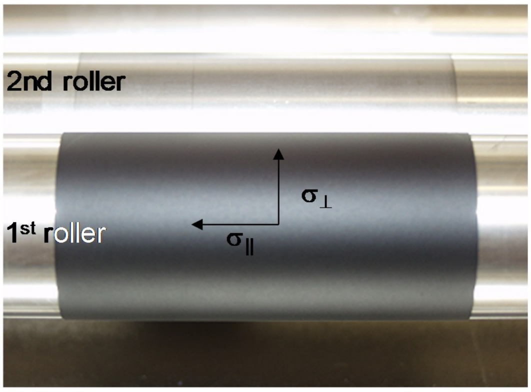

300-100

Figure. S1. Optimization of rolling speed for two-roller process.

If ratio of rolling speed of the 1st roller to the 2nd one was low, surface roughness after rolling process was observed to be large. The optimal ratio of the rolling speeds was set to be 300:100.
